# Estimating Blood Pressure from the Photoplethysmogram Signal and Demographic Features Using Machine Learning Techniques

**DOI:** 10.3390/s20113127

**Published:** 2020-06-01

**Authors:** Moajjem Hossain Chowdhury, Md Nazmul Islam Shuzan, Muhammad E.H. Chowdhury, Zaid B. Mahbub, M. Monir Uddin, Amith Khandakar, Mamun Bin Ibne Reaz

**Affiliations:** 1 Department of Electrical and Computer Engineering, North South University, Dhaka 1229, Bangladesh; moajjem.hossain@northsouth.edu (M.H.C.); nazmul.shuzan@northsouth.edu (M.N.I.S.); 2 Department of Electrical Engineering, Qatar University, Doha 2713, Qatar; amitk@qu.edu.qa; 3 Department of Mathematics and Physics, North South University, Dhaka 1229, Bangladesh; zaid.mahbub@northsouth.edu (Z.B.M.); monir.uddin@northsouth.edu (M.M.U.); 4 Department of Electrical, Electronic & Systems Engineering, Universiti Kebangsaan Malaysia, Bangi Selangor 43600, Malaysia; mamun@ukm.edu.my

**Keywords:** blood pressure, photoplethysmograph, feature selection algorithm, machine learning

## Abstract

Hypertension is a potentially unsafe health ailment, which can be indicated directly from the blood pressure (BP). Hypertension always leads to other health complications. Continuous monitoring of BP is very important; however, cuff-based BP measurements are discrete and uncomfortable to the user. To address this need, a cuff-less, continuous, and noninvasive BP measurement system is proposed using the photoplethysmograph (PPG) signal and demographic features using machine learning (ML) algorithms. PPG signals were acquired from 219 subjects, which undergo preprocessing and feature extraction steps. Time, frequency, and time-frequency domain features were extracted from the PPG and their derivative signals. Feature selection techniques were used to reduce the computational complexity and to decrease the chance of over-fitting the ML algorithms. The features were then used to train and evaluate ML algorithms. The best regression models were selected for systolic BP (SBP) and diastolic BP (DBP) estimation individually. Gaussian process regression (GPR) along with the ReliefF feature selection algorithm outperforms other algorithms in estimating SBP and DBP with a root mean square error (RMSE) of 6.74 and 3.59, respectively. This ML model can be implemented in hardware systems to continuously monitor BP and avoid any critical health conditions due to sudden changes.

## 1. Introduction

Measuring blood pressure (BP) is an important aspect in monitoring the health of a person. High blood pressure, generally, means that a person has a higher risk of health problems [1]. High blood pressure puts a huge amount of strain on the arteries and the heart. This strain can make the arteries less flexible over time. As they become more inflexible, the lumen becomes narrower. Therefore, the probability of it being clogged up (clot) increases. A clot is very dangerous and may cause heart attack, stroke, kidney diseases, and dementia. As a result, it is important for a person to monitor their blood pressure regularly. In most cases, measuring blood pressure once or twice a day is more than enough. However, sometimes the doctor needs to track the blood pressure continuously. This is because blood pressure is known to decrease at night. Therefore, it is useful to measure the blood pressure overnight, as an abnormal dip in blood pressure may suggest a higher risk of cardiovascular problems [2].

The current standard methods include either a cuff-based BP measurement or an invasive procedure for BP measurement. The cuff method measures the blood pressure after a set interval (e.g., of 15 min). This means that the end-result is discrete and uncomfortable to the user. Furthermore, this process requires the arm to be kept steady while the inflation and deflation causes disturbance in the patient’s sleep. Arterial lines management is an invasive procedure that allows for continuous blood pressure monitoring. However, the invasive procedure leaves the patient vulnerable to infection. Hence, there is a need for a noninvasive, cuff-less, continuous BP monitoring system. With the advent of digital sensors, signal-processing, machine learning algorithms and advanced physiological models help gather important human vital signs using wearable sensors [3,4]. Even the indirect estimation of blood pressure (BP) using photoplethysmography (PPG) has become more realistic [5,6,7,8]. 

**Figure 1:**
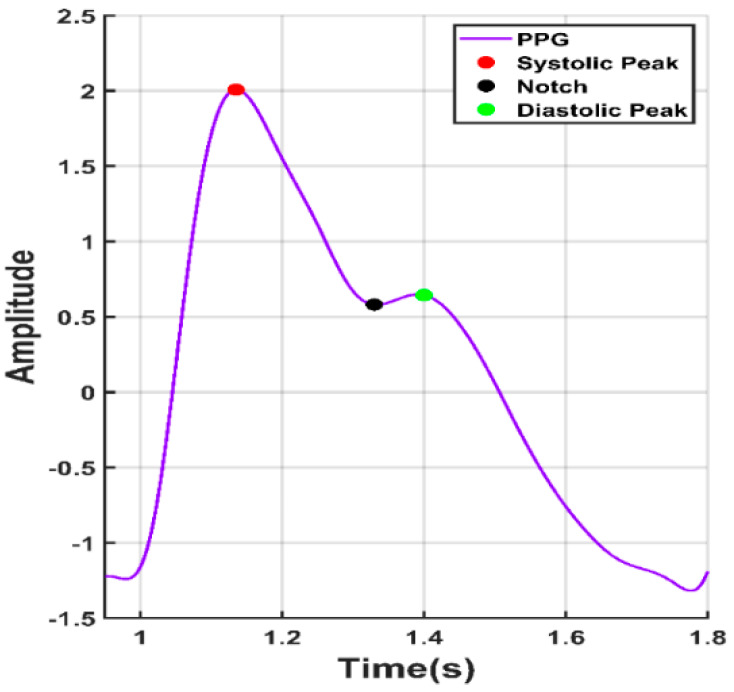
A typical photoplethysmograph (PPG) waveform with notch, systolic peak, and diastolic peak.

Photoplethysmography (PPG) was being used for decades for measuring the amount of light absorbed or reflected by blood vessels in the living tissue. PPG technology is a versatile and low-cost technology [9], which can be extended to different aspects of cardiovascular surveillance including identification of blood oxygen saturation, heart rate, BP estimation, cardiac output, respiration, arterial ageing, endothelial control, micro-vascular blood flow, and autonomic function [10]. Many different kinds of PPG signals have been identified and have been shown associated with age and cardiovascular pathology [11,12]. In clinical practice, PPG signals are recorded from micro-vascular beds at exterior body locations, such as the finger, earlobe, forehead, and toe [13]. The coverage area of the PPG sensor includes veins, arteries, and numerous capillaries. PPG waveforms generally have three distinct features. As shown in Figure 1, a PPG waveform typically contains systolic peak, diastolic peak, and a notch in between.

The raw PPG signal typically includes pulsatile and nonpulsatile blood volumes [14]. The pulsatile portion of the PPG signal is attributed to the variation in blood pressure within the arteries and is synchronous to the pulse, while the nonpulsating part is a result of normal blood volume, respiration, sympathetic nervous system, and thermoregulation [15]. Green, red, and infrared light are often used to extract PPG waveforms. Red and infrared light can reach approximately 2.5 mm, whereas green light can penetrate less than 1 mm into the tissue [16]. Therefore, infrared light is typically used for acquiring the PPG signal for the measurement of blood pressure. Although the PPG tool is a low-cost and portable optical electronic device, its measurement has several challenges, such as, noise reduction [17,18,19] and multi-photodetector creation [20].

Several techniques to estimate BP from PPG were proposed in the recent works. Some algorithms [21] incorporate waveform analysis and biometrics of PPG to estimate BP, which has been tested in subjects with different age, height, and weight. When calibrated, PPG shows great potential to track BP fluctuations, which can bring enormous health and economic benefit. An easy and bio-inspired mathematical model was proposed at [22] to predict estimating systolic BP (SBP) and diastolic BP (DBP) through careful mathematical analysis of the PPG signals. Systolic and diastolic blood pressure levels were predicted using the pulse transit time (PTT) in [23,24] and a combination of paroxysmal atrial tachycardia (PAT) and heart rate in [25], while the combination showed improvement over PTT alone. The beat-to-beat optical BP measurement method was developed, tested, and reported using only PPG from fingertips [26]. Key features such as amplitudes and cardiac part phases were extracted through a fast Fourier transformation (FFT) and used to train an artificial neural network (ANN), which was then used to estimate BP using PPG. In [27], the support vector machine (SVM) algorithm showed better accuracy than the linear regression method and ANN. 

The recent growth in the field of deep learning has made it potential for this application. Su et al., 2018 [28] discussed the problem of accuracy reduction in the current models for BP estimation from PPG due to the requirement of frequent calibration. A deep recurrent neural network (RNN) with long short-term memory (LSTM) was used to create a model for the time-series BP data. PPG and electrocardiogram (ECG) were taken as inputs, and PTT with some other features were used as predictors to estimate BP. This method showed improvements in BP prediction compared to other existing methods. Gotlibovych et al. investigated the potential of using raw PPG data to detect arrhythmia in 2018 [29] with reasonable success, which shows the possibility of using the raw PPG signal as inputs to the deep learners. In [30], the authors have created a novel spectro-temporal deep neural network that took the PPG signal and its first and second derivative as inputs. The neural network model had residual connections and were able to get a mean absolute error (MAE) of 6.88 and 9.43 for DBP and SBP, respectively.

Several research groups have analyzed and evaluated the quality of the open-source dataset, which was used in this study [18,30,31,32]. A novel approach [33] for treating hypertension based on the theory of arterial wave propagation and morphological theory of PPG was proposed to check the physiological changes in different levels of blood pressure. ECG and PPG signals were obtained simultaneously to detect hypertension. A model for PPG characteristic was analyzed and an inherent relationship between the characteristics of systolic BP and PPG was established [34]. In [35], a PPG signal analysis was used to characterize obesity, age group, and hypertension using the PPG pulse based on the pulse decomposition analysis. 

The features typically used for noninvasively estimating BP are: (i) T-domain, (ii) f-domain, (iii) (t,f)-domain, (iv) and statistical features. Several t-domain features, which were calculated from the original signal and its derivatives, were used by different groups [9,36,37,38]. In a different study, Zaid et al. [39] showed the use of frequency domain features for identifying a neurological disorder in this study, the authors have taken inspiration from Zaid et al. to create features in estimating BP accurately from the PPG signal. 

Several studies reported different features of the PPG signal for different applications [9,34,38,40]. Various groups have used these features for SBP and DBP measurement; however, there is still plenty of scope for improvement. Numerous automated ML techniques were evaluated and recorded for various PPG databases as mentioned earlier. Nonetheless, to the best of our knowledge, no recent work has combined t-, f-, and (t,f) domain features to estimate BP with a high accuracy using the machine learning approach. PPG signal processing is comparatively simpler and easier, so more attention is being paid to novel methods that extract features from PPG signals. To reduce the error in BP estimation based on the PPG signal, this analysis not only extracts features from the PPG signal but also utilizes the demographic characteristics of subjects, such as height, weight, and age, etc. There are several features that were extracted for BP estimation from the PPG signal in this study, which were not used before by any other group.

The manuscript is divided into four sections. Section 1 discusses the basics of the PPG signal, related works, and inspirations of this research. The methodology and database are presented in Section 2 along with preprocessing steps and system assessment. Section 3 summarizes and discusses the results while Section 4 concludes the work.

## 2. Materials and Methods

This section discusses the dataset used in the study, the signal preprocessing techniques used, the features extracted, feature selection techniques used, and the machine learning algorithms models trained and tested to estimate SBP and DBP.

**Figure 2:**
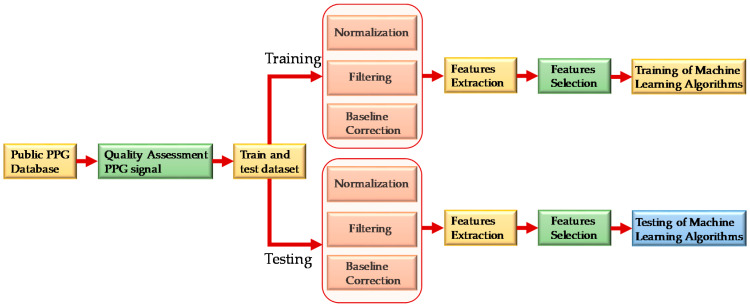
Overall system block diagram.

As shown in Figure 2, PPG signals were first assessed to check signal quality and then randomly divided into two sets. Eighty-five percent of the data was used for training and validation and 15% of the data was used for testing the performance of the model. The PPG signals were preprocessed before they were sent for feature extraction. After extracting meaningful features, feature selection techniques were used to reduce computational complexity and the chance of over-fitting the algorithm. The features were then used to train machine learning algorithms. The best regression model was selected for SBP and DBP estimation individually.

### 2.1. Dataset Description

The dataset used in this study was taken from Liang et al. [31], which is publicly available. The dataset contained 657 PPG signal samples from 219 subjects [18]. The PPG signal were sampled at a rate of 1000 Hz and contained 2100 data points per signal with a signal duration of 2.1 s. Other than the PPG signal, the patient’s demographic information such as age, gender, height, and weight along with systolic pressure, diastolic pressure, and heart rate were also recorded. A summary of the dataset is shown in Table 1.

**Figure 3:**
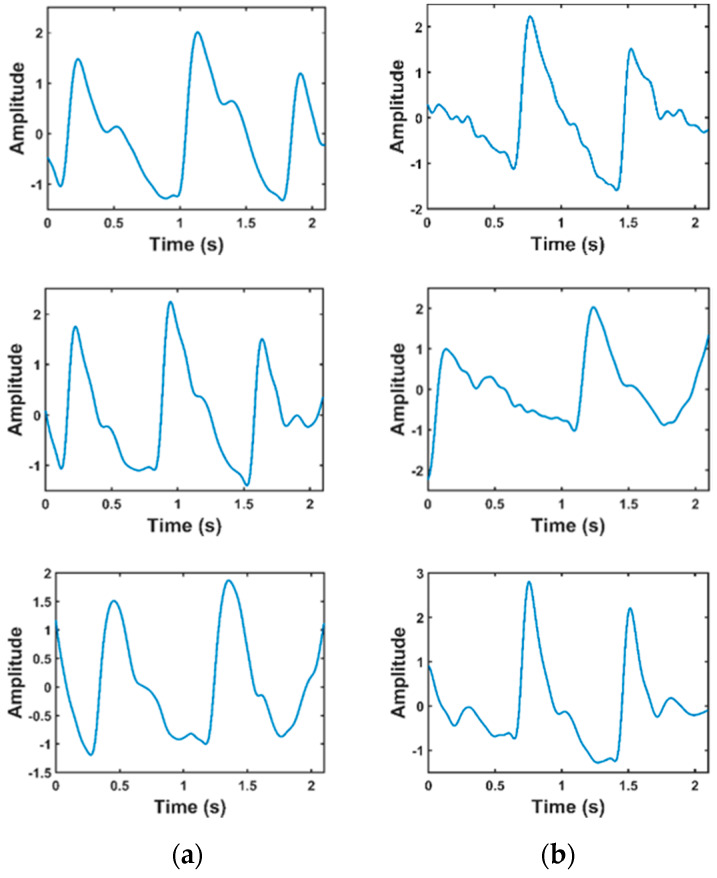
Comparison of waveforms that are fit and unfit for the study. (**a**) Fit data; (**b**) unfit data.

Of the 657 signals, many signals were of poor quality and could not be used for feature extraction. Liang et al. [18] used a skewness-based signal quality index (SQI) to find the suitable signals. In the quality assurance process, 222 signals from 126 subjects were finally kept for this study. Figure 3 shows the sample PPG signal which were divided as fit and unfit for the study. It is obvious that the unfit waveforms either do not have prominent features or the diastolic part of the waveform is not obvious in the recorded signal and the data length is very short. Hence, they were not used for the study.

### 2.2. Preprocessing Signals

The raw PPG signals were prepared through different preprocessing stages before feature extraction, which are summarized below.

#### 2.2.1. Normalization

To extract meaningful information from the signals, it was necessary to normalize all the signals. The Z-score technique was used to normalize the signals in this study to get amplitude-limited data.

(1) $$Z-\mathrm{score}\;\mathrm{Normalized}\;\mathrm{Signal}=\frac{\mathrm{Signal}-\mathrm{Signal}\;\mathrm{Mean}}{\mathrm{Standard}\;\mathrm{Deviation}\;\mathrm{of}\;\mathrm{Signal}}$$

**Figure 4:**
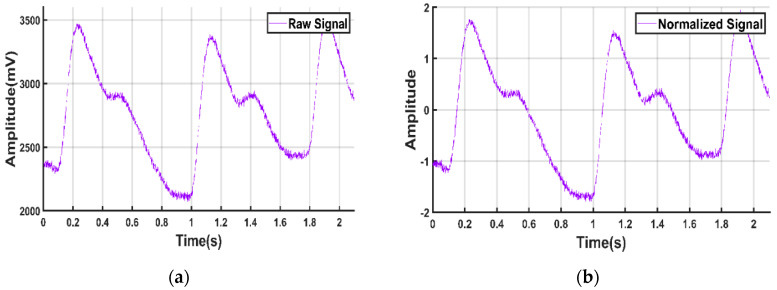
PPG signal. (**a**) Before normalization; (**b**) after normalization.

It was also observed that after normalization, other preprocessing techniques were easier to implement. Figure 4 shows the sample PPG signal before and after normalization.

#### 2.2.2. Signal Filtration

**Figure 5:**
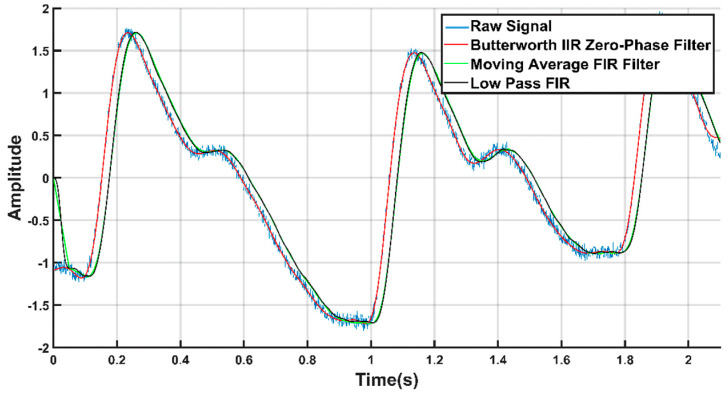
Filtered signals overlaid on the raw PPG signal.

It was observed that, the signal from the database [31] has high-frequency noise components. Thus, the signals were filtered through a low-pass filter that can remove these high-frequency components. Several filtration techniques were tested to denoise the signal, such as, moving average, low pass finite impulse response (FIR), and Butterworth infinite impulse response (IIR) zero-phase filter. Figure 5 shows the raw signal overlaid with the filtered output using different type of filters. From Figure 5, we can see that the Butterworth filter produced the filtration. Hence, we used it to filter the PPG waveforms, which was also used by others to remove noise from the PPG signals [9,12,37,41]. In this work, the sixth order IIR filter with a cut-off frequency of 25 Hz was designed in MATLAB.

#### 2.2.3. Baseline Correction

**Figure 6:**
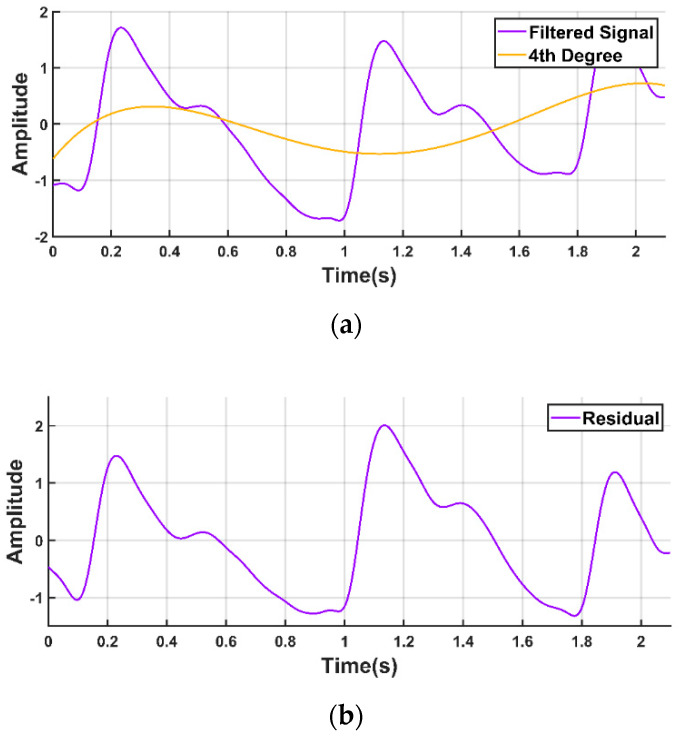
Baseline correction of PPG waveform. (**a**) PPG waveform with the baseline wandering and fourth degree polynomial trend; (**b**) PPG waveform after detrending.

The PPG waveform is commonly contaminated with a baseline wandering due to respiration at frequencies ranging from 0.15 to 0.5 Hz [11,21,42,43]. Therefore, it is very important that the signal is properly filtered to remove the baseline wandering but that important information is preserved as far as possible. We used a polynomial fit to find the trend in the signal. Then, we subtracted the trend to get the baseline corrected signal, as shown in Figure 6.

### 2.3. Feature Extraction

**Figure 7:**
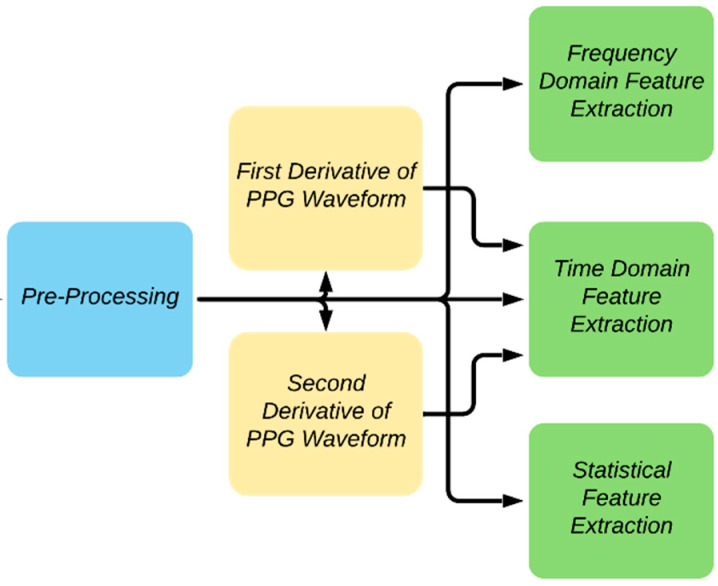
Overview of feature extraction.

The block diagram summarizing the feature extraction details adopted in the study is shown in Figure 7. A PPG waveform contains many informative information such as systole, diastole, notch, pulse width, peak-to-peak interval, etc. Some of the distinctive features of the PPG waveform might not be dominant in some patients, such as the notch prevalence changing with age [44]. To find the different key points of the PPG signal, the authors have followed the methods described in the previous work [45]. The technique was largely based on the derivatives and thresholds defined in [46] and [47].

**Figure 8:**
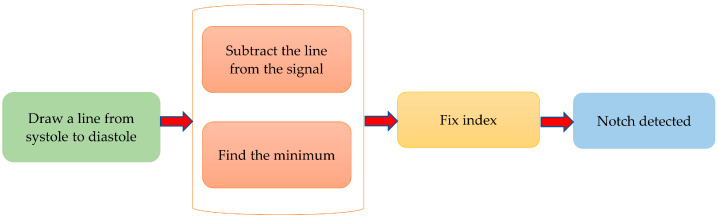
Algorithm of notch detection.

**Figure 9:**
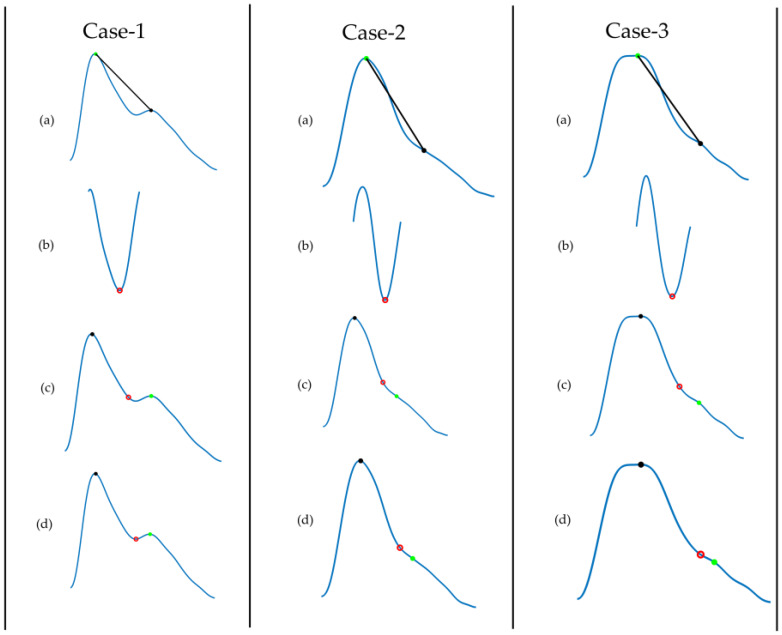
Demonstration of dicrotic notch detection for different age groups: Case 1 (26 years), 2 (45 years), and 3 (80 years). (**a**) Filtered PPG signal where we draw a line from systolic peak to diastolic peak; (**b**) subtract the line from the signal and find its minimum point; (**c**) initial notch detected; (**d**) adjust the notch using the fix index.

The dicrotic notch is an essential feature of the PPG signal. Figure 8 describes the algorithm to detect the dicrotic notch. To do so, a line was drawn from the systolic peak to the diastolic peak. The minimum of the subtraction of the straight line from the signal is the dicrotic notch. However, to make it more robust, the fix index was used, which calculates the local minima within a given window (in this case 50 ms) around a given point. Reliable detection of the dicrotic notch in various situations is shown in Figure 9.

**Figure 10:**
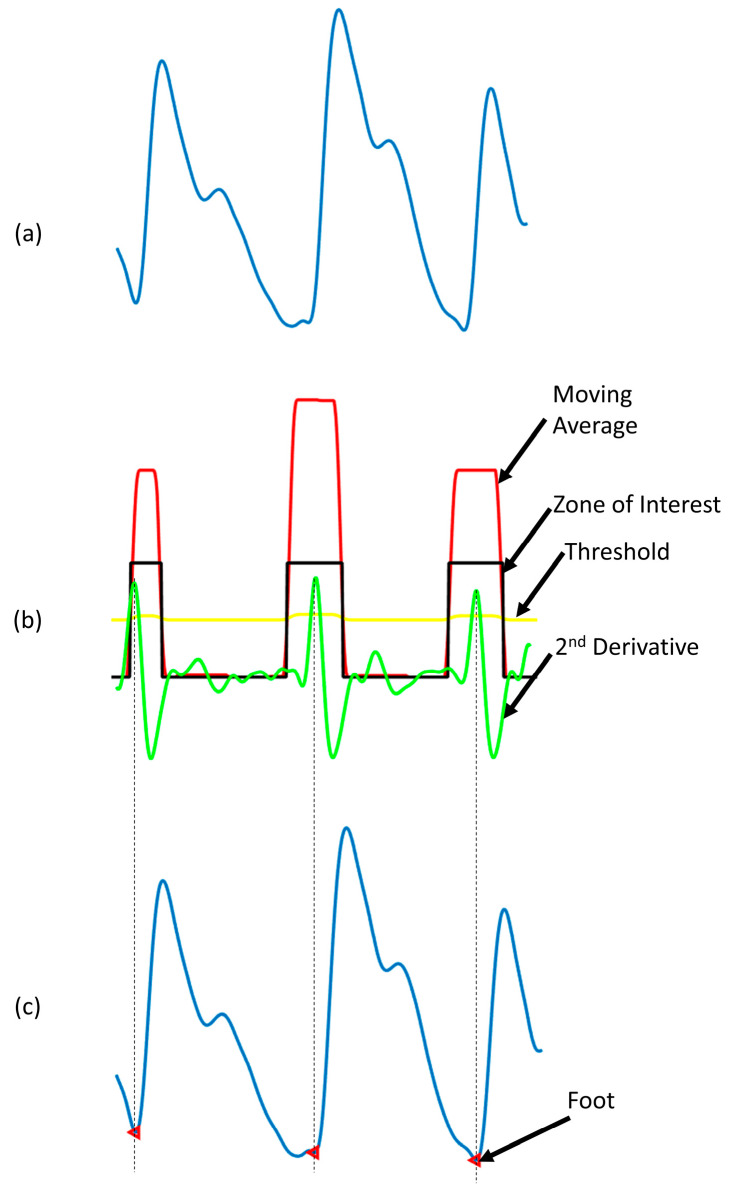
Detection of the foot of a PPG waveform. (**a**) Filtered PPG signal; (**b**) second derivative of PPG along with derivation of the zone of interest based on moving average of acceleration plethysmogram (APG) and adaptive threshold; (**c**) foot of the signal detected.

Another key feature is the foot of the PPG signal. To find the foot of the PPG waveform, the second derivative of the PPG waveform, also called acceleration plethysmogram (APG) was first calculated. From the APG, a zone of interest was defined, where the moving average of APG is larger than an adaptive threshold. In the zone of interest, the highest point of the APG corresponds to the foot of the signal. This method is robust and allows detecting the foot of the signal very accurately. Figure 10 shows that the algorithm can detect the prominent foot and flat foot accurately [45].

**Figure 11:**
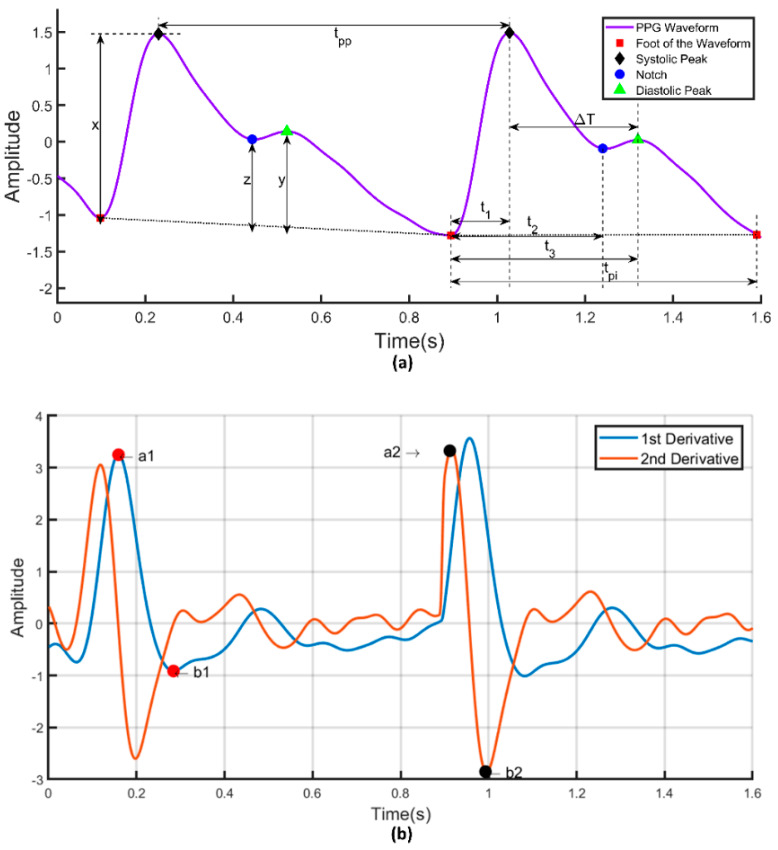
(**a**) Illustration of time-domain features in a PPG signal. (**b**) First and second derivatives of PPG signal.

**Figure 12:**
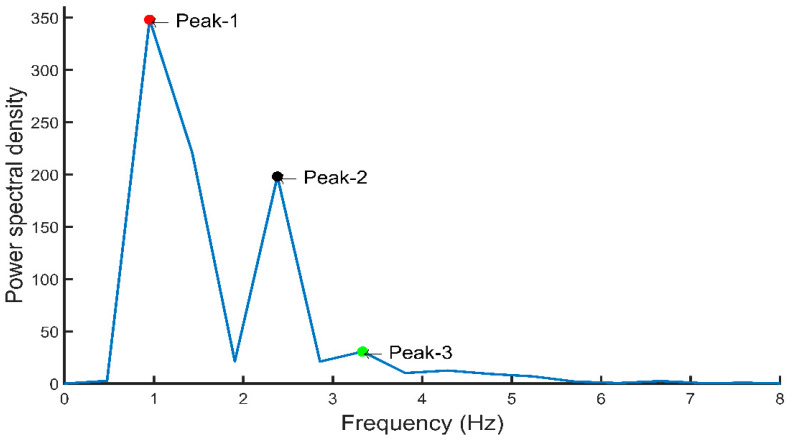
Frequency-domain representation of PPG signal with important features.

PPG signal’s first and second derivatives were calculated and the relationship between PPG signals and their first and second derivatives is shown in Figure 11. The PPG signal is analyzed to extract the a1 and b1 point from its first derivative as well as the a2 and b2 point from the second derivative. Figure 12 shows the frequency domain representation of the PPG signal. The frequency domain representation was analyzed and features related to the first three peaks were extracted. The length of the fast Fourier transform was 2100, which was equal to the number of data points in the signal. Furthermore, demographic data such as height, weight, BMI, gender, age, and heart rate were also used as features. It was reported by several groups that demographic features are important features for BP estimation [48]. Elgendi [9] emphasized the need of height details for accurate estimation of the PPG waveform while Kavasaoglu et al. [36] found that demographic features were useful and highly ranked features in their machine learning algorithm using PPG signal’s characteristics features. In a real-time scenario, age and BMI will be known to the user and the heart rate can be easily calculated from the PPG signal. Definitions of the extracted time-domain and demographic features were listed in Table 2, Table 3, Table 4 and Table 5. Frequency-domain and statistical features can significantly contribute to BP estimation and were defined in Table 6, Table 7 and Table 8 respectively. Therefore, 107 features encompassing seventy-five t-domain, sixteen f-domain, and ten statistical features were derived for each PPG signal along with six demographic data. The t-domain, f-domain, and statistical features were identified from different previous works [3,4,9,23,25,26,27,38,39]. It is reported in the literature that 1–24 and 42–58 features were used in PPG related works [49]. These features are considered as literature features in Section 3.

### 2.4. Feature Selection

Feature selection or reduction is important to reduce the risk of over-fitting the algorithms. In this work, three feature selection methods: Correlation-based feature selection (CFS), ReliefF features selection [50], and features for classification using the minimum redundancy maximum relevance (fscmrmr) algorithm. ReliefF is a feature selection algorithm, which randomly selects instances and adjusts the weights of the respective element depending on the nearest neighbor [51].

Correlation is a test used to evaluate whether or not a feature is highly correlated with the class or not highly correlated with any of the other features [52,53]. On the other hand, the fscmrmr algorithm finds an optimal set of features that are mutually and as dissimilar as possible, and can effectively represent the response variable. The algorithm minimizes a feature set’s inconsistency and maximizes the relevance of a feature set to the answer variable [54]. MATLAB built-in functions were used for CFS, ReliefF, and fscmrmr feature selection algorithm [55]. 

In Table 9, the features selected by the feature reduction algorithm are listed. The features listed are those that produced the best results.

### 2.5. Machine Learning (ML) Algorithms

After the features were extracted, the feature matrix was trained with machine learning algorithms. The Regression Learner App of MATLAB 2019b was used to estimate the BP. Five different algorithms (linear regression, regression trees, support vector regression (SVR), Gaussian process regression (GPR), and ensemble trees) with their variations to a total of 19 algorithms were trained using the 10-fold cross validation. Out of all these algorithms, two best performing algorithms, Gaussian process regression and ensemble trees were tested. 

1. Gaussian Process Regression: GPR is a nonparametric Bayesian regression approach [56], which has benefits of operating well on small datasets and being able to provide measures of uncertainty on the predictions. Unlike many common supervised machine learning algorithms that learn the exact values in a function for each parameter, the Bayesian approach infers a distribution of probability over all possible values.

2. Ensemble Trees: An ensemble tree is a predictive model consisting of a weighted combination of multiple regression trees [57]. The core idea behind the ensemble model is to pull together a set of weak learners to create a strong learner.

### 2.6. Hyper-Parameters Optimization of the Best Performing Algorithm

The machine learning algorithms used were initially trained with default parameters. The performance of these algorithms can, however, be improved by optimizing their hyper-parameters. Hyper-parameters optimization was carried out on the algorithms using the MATLAB 2019b Regression Learner App [58].

### 2.7. Evaluation Criteria

To evaluate the performance of the ML algorithms for estimating BP, four criteria were used. Here, Xp is the predicted data while the ground truth data is X and n is the number of samples:

1. Mean Absolute Error (MAE): Absolute error is the amount of predicted error. The mean absolute error is the mean of all absolute errors.

(2) $$\mathrm{MAE}=\frac{1}{n}\sum_{n}\left|X_{p}-X\right|$$

2. Mean Squared Error (MSE): MSE calculates the squared sum of the errors. MSE is a risk function, which corresponds to the expected value of the squared error loss. MSE contains both the estimator’s variance and its bias.

(3) $$\mathrm{MSE}=\frac{\sum^{\;}{\left|X_{p}-X\right|}^{2}}{n}$$

3. Root Mean Squared Error (RMSE): RMSE is the standard deviation of the residuals (prediction error). Residuals are a measure of how far away the data points are from the regression line; RMSE is a measure of how these residuals are spread out.

(4) $$\mathrm{RMSE}=\sqrt{\frac{\sum^{\;}{\left|X_{p}-X\right|}^{2}}{n}}=\sqrt{\mathrm{MSE}}$$

4. Correlation Coefficient (R): It is a statistical technique, which measures how closely related are two variables (predictors and the predictions). It also tells us how close the predictions are to the trendline.

(5) $$R=\sqrt{1-\frac{\mathrm{MSE}\left(\mathrm{Model}\right)}{\mathrm{MSE}\left(\mathrm{Baseline}\right)}}\;\mathrm{where}\;\mathrm{MSE}\;(\mathrm{Baseline})=\frac{\sum^{\;}{\left|X-\mathrm{mean}\left(X\right)\right|}^{2}}{n}$$

When using the Regression Learner App in MATLAB, the above criteria are automatically calculated by MATLAB and these values were used to evaluate the performance of the algorithms. Among these criteria, RMSE was chosen as the main criterion.

## 3. Results and Discussion

This section summarizes the performance of the machine learning algorithm used in the study. As stated earlier, 19 different machine learning algorithms were trained and validated. It is observed from Table 9 that the features of Table 5 have a significant contribution along with demographic features in estimation. Out of the 19 algorithms, GPR and ensemble trees outperformed for all cases in the estimation of both systolic blood pressure and diastolic blood pressure.

**Figure 13:**
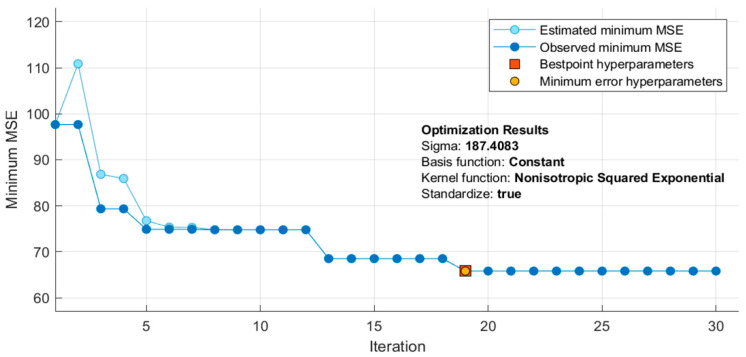
Optimization of the Gaussian process regression (GPR) model during training.

In Table 10, it can be noticed that the ReliefF feature selection algorithm produced the best result when combined with GPR. The feature selected using a combination of ReliefF and GPR performed the best in estimating SBP while CFS and GPR performed best for DBP. Moreover, R scored 0.74 and 0.68 for SBP and DBP, respectively, which means that there is a strong correlation with the predictors and the ground truth. However, these results could be further improved by tuning the hyper-parameters. The Bayesian optimization was used, which is efficient and effective and operates by constructing a probabilistic model of the objective function, called the surrogate function, which is then optimally scanned with the acquisition function before the candidate samples are selected for evaluation of the real objective function. As shown in Figure 13, 30 iterations of the model were trained during optimization. Each time it iterates, it tunes the hyper-parameters. If the result gives an MSE, lower than the lowest MSE recorded, then that MSE is taken as the lowest. If there is no over-fitting, the lowest MSE should be reported at the end of the iterations. 

**Figure 14:**
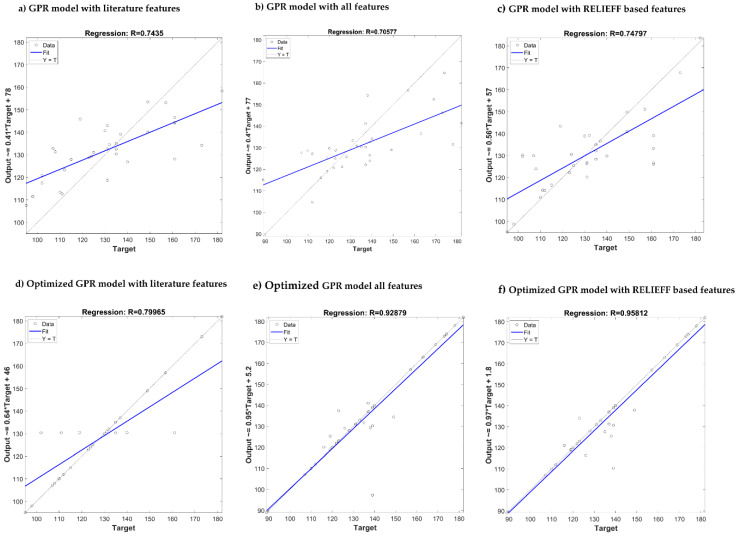
Comparison of the predicted output vs. actual target for SBP estimation using different GPR: (**a**–**c**) Models without optimization, (**d**–**f**) models with optimization.

**Figure 15:**
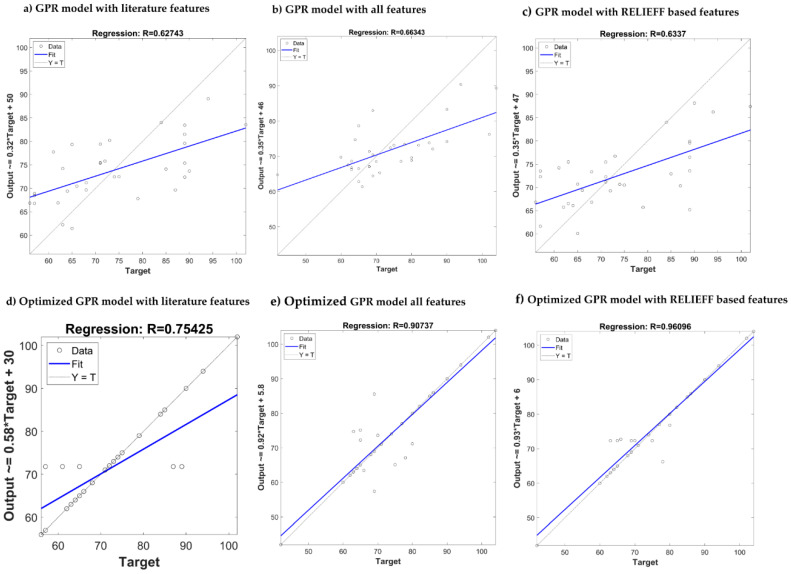
Comparison of the predicted output vs. actual target for DBP estimation using different GPR: (**a**–**c**) Models without optimization, (**d**–**f**) models with optimization.

Table 11 summarizes the performances of the algorithms after optimization. It is clear that the ReliefF feature selection algorithm with GPR outperforms the other algorithms. After optimization, the combination produced a remarkable improvement in R score for SBP and DBP estimation (0.95/0.96). Comparison of the predicted output with the actual target has been shown in Figure 14 and Figure 15 for SBP and DBP respectively. Three of the best performing models are shown along with their results after the model has been optimized. In both Figure 14 and Figure 15, optimizing ReliefF based model produced the best fit.

In general, due to different evaluation criteria, and different and inadequately defined datasets, it is difficult to compare similar works in this field. Some reported lowest errors using small selected subsets of public or private data, but others worked on large-scale data (Kachuee et al. [24] and Slapničar et al. [30]) which has greater errors. Looking at individual related works in Table 12, Kachuee et al. [24] proposed a method that employs physiological parameters, machine learning, and signal processing algorithms using the PTT approach and some time-domain PPG features, where they showed a promising result according to the British Hypertension Society (BHS). Kim et al. [23] compared the artificial neural network (ANN) with multiple regressions as a BP estimation method, but their study is limited to 20 subjects only and did not identify DBP. Cattivelli et al. [25] introduced an algorithm for estimating BP, but used a very small amount of data (34 recordings for 25 subjects). Zhang et al. [27] described the SVM and neural network approach using time-domain features, which is used directly for the study of BP regression, and good results were obtained compared to the previous work.

In [59], Zadi et al. showed the calculation of systolic and diastolic BP from PPG measurements using a viable method for continuous and noninvasive measurement of BP, however, using a very small dataset (15 subjects only). Slapničar et al. [30] worked with a large dataset and using the deep-learning spectro-temporal ResNet algorithm has achieved a reasonable accuracy in estimation. Su et al. [28] used a conventional deep learning model for LSTM, but used the PTT approach as opposed to using only PPG on a small database. Finally, using time-domain, frequency-domain, and statistical features to train an optimized feature reduced regression model, a very low error rate was achieved in this work. To the best of our knowledge, no work has extracted all these features and achieved such an error rate using the classical machine learning approach. In Table 12, a comparative summary of recent works with this work is shown in respect to the evaluation parameters: MAE, MSE, RMSE, and R.

It is also important to note that the standard for the evaluation of blood pressure measurement devices proposed by the Association for the Advancement of Medical Instrumentation (AAMI), the British Hypertension Society (BHS), and the International Organization for Standardization [60,61,62,63] is that a device is considered acceptable if the estimated blood pressure is less than 10 mmHg from the actual. The machine learning algorithm proposed in the study was estimated with much higher precision and accuracy. According to Table 13, the AAMI standard completely accepts the results of the GPR algorithm in DBP. However, the SD (standard deviation) of the model in the SBP evaluation is greater than the standard’s maximum permissible range, but the mean is well in the acceptable range. 

In addition, the accuracy of the proposed algorithm is tested from the point of view of the BHS grading criteria. Grades represent the cumulative percentage of readings falling within 5, 10, and 15 mmHg of the mercury standard. The GPR algorithm findings are shown in Table 14, based on the BHS standard. The GPR model performance is consistent with the BHS standard grade B for both SBP and DBP estimation.

## 4. Conclusions

In this study, the authors have proposed and implemented a method for estimating systolic and diastolic blood pressure with the help of PPG signal features and machine learning algorithms. This successfully demonstrates how the PPG signal can be used to accurately estimate the BP of patients noninvasively without using a cuff-based pressure measurement. The entire preprocessing method of the PPG fingertip signals to extract the features, feature reduction, and training of the algorithms were discussed. The raw signals were treated in different techniques and the resulting waveform has a high signal-to-noise ratio and is free from baseline wandering. The system used time-domain, frequency-domain, and statistical features along with demographic data adding up to 107 features, to extract meaningful data. Models for SBP and DBP were trained separately as they often had different key features. Nineteen different machine learning algorithms were trained for both SBP and DBP, out of which GPR and ensemble trees were the most promising. To reduce computational complexity, various feature selection methods were used. It was found that a combination of ReliefF feature selection and GPR machine learning algorithm produced the best results. However, hyper-parameter optimization was then used to improve the models further. The resulting models achieved a noteworthy R score of 0.95 and 0.96 for SBP and DBP, respectively. The DBP estimator fulfills the requirement of the AAMI standard while the SBP estimator is following the mean requirement but falls short than the standard deviation requirement by a small amount. SBP and DBP both fulfill the grade B criteria according to the BHS standard. In the future work, deep learning algorithms can be utilized with a larger dataset to produce a better prediction model, which can fulfill the A grade requirement of the BHS standard. The trained model can be used in developing commercial light computation-based prototypes that can accurately estimate the BP. Such a system can help in continuously monitoring BP and avoiding any critical health conditions due to sudden changes.

## Figures and Tables

**Table 1:** Data summary.

Physical Index	Numerical Data
Females	115 (52%)
Age (years)	57 ± 15
Height (cm)	161 ± 8
Weight (kg)	60 ± 11
Body Mass Index (kg/m^2^)	23 ± 4
Systolic Blood Pressure (mmHg)	127 ± 20
Diastolic Blood Pressure (mmHg)	71 ± 11
Heart Rate (beats/min)	73 ± 10

**Table 2:** Twenty-four features from the PPG signal.

Feature	Definition
1. Systolic Peak	The amplitude of (‘x’) from PPG waveform
2. Diastolic Peak	The amplitude of (‘y’) from PPG waveform
3. Height of Notch	The amplitude of (‘z’) from PPG waveform
4. Systolic Peak Time	The time interval from the foot of the waveform to the systolic peak (‘t_1_^’^)
5. Diastolic Peak Time	The time interval from the foot of the waveform to the height of notch (‘t_2_^’^)
6. Height of Notch Time	The time interval from the foot of the waveform to the diastolic peak (‘t_3_^’^)
7. ∆T	The time interval from systolic peak time to diastolic peak time
8. Pulse Interval	The distance between the beginning and the end of the PPG waveform (‘t_pi_^’^)
9. Peak-to-Peak Interval	The distance between two consecutive systolic peaks (t_pp_)
10. Pulse Width	The half-height of the systolic peak
11. Inflection Point Area	The waveform is first split into two parts at the notch point. The area of the first part is A_1_ and the area of the second part is A_2_. The ratio of A_1_ and A_2_ is the inflection point area (‘A_1_/A_2_ ’)
12. Augmentation Index	The ratio of diastolic and systolic peak amplitude (‘y/x’)
13. Alternative Augmentation Index	The difference between systolic and diastolic peak amplitude divided by systolic peak amplitude (‘(x-y)/x’)
14. Systolic Peak Output Curve	The ratio of systolic peak time to systolic peak amplitude (‘t_1/_x’)
15. Diastolic Peak Downward Curve	The ratio of diastolic peak amplitude to the differences between pulse interval and height of notch time (‘y/ t_pi_-t_3_’)
16. t_1_/t_pp_	The ratio of systolic peak time to the peak-to-peak interval of the PPG waveform
17. t_2_/t_pp_	The ratio of notch time to the peak-to-peak interval of the PPG waveform
18. t_3_/t_pp_	The ratio of diastolic peak time to the peak-to-peak interval of the PPG waveform
19. ∆T/t_pp_	The ratio of ∆T to the peak-to-peak interval of the PPG waveform
20. z/x	The ratio of the height of notch to the systolic peak amplitude
21. t_2_/z	The ratio of the notch time to the height of notch
22. t_3_/y	The ratio of the diastolic peak time to the diastolic peak amplitude
23. x/(t_pi_-t_1_)	The ratio of systolic peak amplitude to the difference between pulse interval and systolic peak time
24. z/(t_pi_-t_2_)	The ratio of the height of notch to the difference between pulse interval and notch time

**Table 3:** Seventeen width-related PPG features.

Feature	Definition
25. Width (25%)	The width of the waveform at 25% amplitude of systolic amplitude
26. Width (75%)	The width of the waveform at 75% amplitude of systolic amplitude
27. Width (25%)/t1	The ratio of pulse width at 25% of systolic amplitude to systolic peak time
28. Width (25%)/t2	The ratio of pulse width at 25% of systolic amplitude to notch time
29. Width (25%)/t3	The ratio of pulse width at 25% of systolic amplitude to diastolic peak time
30. Width (25%)/∆T	The ratio of pulse width at 25% of systolic amplitude to ∆T
31. Width (25%)/tpi	The ratio of pulse width at 25% of systolic amplitude to pulse interval
32. Width (50%)/t1	The ratio of pulse width at 50% of systolic amplitude to systolic peak time
33. Width (50%)/t2	The ratio of pulse width at 50% of systolic amplitude to notch time
34. Width (50%)/t3	The ratio of pulse width at 50% of systolic amplitude to diastolic peak time
35. Width (50%)/∆T	The ratio of pulse width at 50% of systolic amplitude to ∆T
36. Width (50%)/tpi	The ratio of pulse width at 50% of systolic amplitude to pulse interval
37. Width (75%)/t1	The ratio of pulse width at 75% of systolic amplitude to systolic peak time
38. Width (75%)/t2	The ratio of pulse width at 75% of systolic amplitude to notch time
39. Width (75%)/t3	The ratio of pulse width at 75% of systolic amplitude to diastolic peak time
40. Width (75%)/∆T	The ratio of pulse width at 75% of systolic amplitude to ∆T
41. Width (75%)/tpi	The ratio of pulse width at 75% of systolic amplitude to pulse interval

**Table 4:** Sixteen features derived from the first and second derivative.

Feature	Definition
42. a_1_	The first maximum peak from the first derivative of the PPG waveform
43. t_a1_	The time interval from the foot of the PPG waveform to the time at which a_1_ occurred
44. a_2_	The first maximum peak from the second derivative of the PPG waveform after a_1_
45. t_a2_	The time interval from the foot of the PPG waveform to the time at which a_2_ occurred
46. b_1_	The first minimum peak from the first derivative of the PPG waveform after a_1_
47. t_b1_	The time interval from the foot of the PPG waveform to the time at which b_1_ occurred
48. b_2_	The first minimum peak from the second derivative of the PPG waveform after a_2_
49. t_b2_	The time interval from the foot of the PPG waveform to the time at which b_2_ occurred
50. b_2_/a_2_	The ratio of b_2_ to a_2_
51. b_1_/a_1_	The ratio of first minimum peak of the first derivative after a_1_ to first maximum peak of the first derivative
52. t_a1_/t_pp_	The ratio of t_a1_ to the peak-to-peak interval of the PPG waveform
53. t_b1_/t_pp_	The ratio of t_b1_ to the peak-to-peak interval of the PPG waveform
54. t_b2_/t_pp_	The ratio of t_b2_ to the peak-to-peak interval of the PPG waveform
55. t_a2_/t_pp_	The ratio of t_a2_ to the peak-to-peak interval of the PPG waveform
56. (t_a1–_t_a2_)/t_pp_	The ratio of the difference between t_a1_ and t_a2_ to the peak-to-peak interval of the PPG waveform
57. (t_b1–_t_b2_)/t_pp_	The ratio of the difference between t_b1_ and t_b2_ to the peak-to-peak interval of the PPG waveform

**Table 5:** Eighteen demographic time-domain features.

Feature	Definition
58. Height/∆T	It is known as stiffness index
59. Weight/∆T	The ratio of weight to ∆T
60. BMI/∆T	The ratio of BMI to ∆T
61. Height/t_1_	The ratio of height to the systolic peak time
62. Weight/t_1_	The ratio of weight to the systolic peak time
63. BMI/t_1_	The ratio of BMI to the systolic peak time
64. Height/t_2_	The ratio of height to the notch time
65. Weight/t_2_	The ratio of weight to the notch time
66. BMI/t_2_	The ratio of BMI to the notch time
67. Height/t_3_	The ratio of height to the diastolic peak time
68. Weight/t_3_	The ratio of weight to the diastolic peak time
69. BMI/t_3_	The ratio of BMI to the diastolic peak time
70. Height/t_pi_	The ratio of height to the pulse interval
71. Weight/t_pi_	The ratio of weight to the pulse interval
72. BMI/t_pi_	The ratio of BMI to the pulse interval
73. Height/t_pp_	The ratio of height to the peak-to-peak interval
74. Weight/t_pp_	The ratio of weight to the peak-to-peak interval
75. BMI/t_pp_	The ratio of BMI to the peak-to-peak interval

**Table 6:** Sixteen frequency-domain features.

Feature	Definition
76. Peak-1	The amplitude of the first peak from the fast Fourier transform of the PPG signal
77. Peak-2	The amplitude of the second peak from the fast Fourier transform of the PPG signal
78. Peak-3	The amplitude of the third peak from the fast Fourier transform of the PPG signal
79. Freq-1	The frequency at which the first peak from the fast Fourier transform of the PPG signal occurred
80. Freq-2	The frequency at which the second peak from the fast Fourier transform of the PPG signal occurred
81. Freq-3	The frequency at which the third peak from the fast Fourier transform of the PPG signal occurred
82. A0–2	Area under the curve from 0 to 2 Hz for the fast Fourier transform of the PPG signal
83. A2–5	Area under the curve from 2 to 5 Hz for the fast Fourier transform of the PPG signal
84. A0–2/A2–5	The ratio of the area under the curve from 0 to 2 Hz to the area under the curve from 2 to 5 Hz
85. Peak-1/peak-2	The ratio of the first peak to the second peak from the fast Fourier transform of the PPG signal
86. Peak-1/peak-3	The ratio of the first peak to the third peak from the fast Fourier transform of the PPG signal
87. Freq-1/freq-2	The ratio of the frequency at first peak to the frequency at second peak from the fast Fourier transform of the PPG signal
88. Freq-1/freq-3	The ratio of the frequency at first peak to the frequency at third peak from the fast Fourier transform of the PPG signal
89. Maximum Frequency	The value of highest frequency in the signal spectrum $f_{max}$
90. Magnitude at Fmax	Signal magnitude at highest frequency X$\left(f_{max}\right)$
91. Ratio of signal energy	Ratio of signal energy between $(f_{max}\pm \Delta f$) and the whole spectrum X$(f_{max}\pm \Delta f)/\sum_{i=0}^{N-1}X_{i}\left(f\right)$

**Table 7:** Ten statistical features.

Feature	Definition	Equation
92. Mean	Sum of all data divided by the number of entries	$\bar{x}=\frac{\sum^{\;}x}{n}$
93. Median	Value that is in the middle of the ordered set of data	Odd numbers of entries: Median = middle data entry. Even numbers of entries: Median = adding the two numbers in the middle and dividing the result by two.
94. Standard Deviation	Measure variability and consistency of the sample.	s = $\sqrt{\frac{\sum^{\;}x-\bar{x}}{n-1}}$
95. Percentile	The data value at which the percent of the value in the data set are less than or equal to this value.	25th = ($\frac{25}{100}$)n
		75th = ($\frac{75}{100}$)n
96. Mean Absolute Deviation	Average distance between the mean and each data value.	MAD = $\frac{\sum_{i=1}^{n}\left|\;x_{i}-\bar{x\;}\right|}{n}$
97. Inter Quartile Range (IQR)	The measure of the middle 50% of data.	IQR = Q_3_–Q_1_ Q_3_: Third quartile, Q_1_: First quartile, Quartile: Dividing the data set into four equal portions.
98. Skewness	The measure of the lack of symmetry from the mean of the dataset.	g1 = $\frac{\sum_{i=1}^{N}{\left(Y_{i}-Y\right)}^{3}/N}{S^{3}}$ Y: Mean, s: Standard deviation, N: Number of data.
99. Kurtosis	The pointedness of a peak in distribution curve, in other words it is the measure of sharpness of the peak of distribution curve.	K = $\frac{\sum_{i=1}^{N}{\left(Y_{i}-Y\right)}^{4}/N}{S^{4}}-3$ Y: Mean, s: Standard deviation, N: Number of data.
100. Shannon’s Entropy	Entropy measures the degree of randomness in a set of data, higher entropy indicates a greater randomness, and lower entropy indicates a lower randomness.	H(x) = −$\sum_{i=0}^{N-1}p_{i}\log_{2}p_{i}$
101. Spectral Entropy	The normalized Shannon’s entropy that is applied to the power spectrum density of the signal.	SEN =$\;\frac{-\sum_{i=0}^{N-1}p_{k\log_{2}p_{k}}}{\log N}$ $p_{k}$: Spectral power of the normalized frequency, N: Number of frequencies in binary

**Table 8:** Six demographic features.

102. Height	103. Weight	104. Gender	105. Age	106. BMI	107. Heart rate

**Table 9:** Features chosen by the feature selection algorithms.

Feature Selection Algorithms Used	Systolic Blood Pressure	Diastolic Blood Pressure
RELIEFF	105. Age, 106. Heart Rate, 103. Weight, 102. Height, 107. BMI, 83. A2–5, 63. BMI/t_1_, 71. Weight/t_pi_, 74. Weight/t_pp_, 62. Weight/t_1_, 75. BMI/t_pp_	105. Age, 106. Heart Rate, 103. Weight, 102. Height, 107. BMI, 69. BMI/t_3_, 71. Weight/t_pi_, 6. t_3_, 72. BMI/t_pi_, 82. A0–2,
FSCMRMR	105. Age, 97. Inter Quartile Range, 45. t_a2_, 64. Height/t_2_, 13. Alternative Augmentation Index, 98. Skewness, 101. Spectral Entropy, 87. Freq-1/Freq-2, 23. x/(t_pi_-t_1_), 32. Width (50%)/t1, 36. Width (50%)/tpi, 99. Kurtosis, 30. Width (25%)/∆T	103. Weight, 22. t_3_/y, 106. Heart Rate, 40. Width (75%)/∆T, 77. Peak-2, 100. Shannon’s Entropy, 96. Mean Absolute Deviation, 90. Magnitude at Fmax, 38. Width (75%)/t_2_, 58. Height/∆T, 101. Spectral Entropy, 31. Width (25%)/tpi, 105. Age
CFS	69. BMI/t_3_, 71. Weight/t_pi_, 74. Weight/t_pp_, 49. t_b2_, 59. Weight/∆T, 51. b_1_/a_1_, 46. b_1_, 47. t_b1_, 62. Weight/t_1_, 52. t_a1_/t_pp_, 66. BMI/t_2_, 67. Height/t_3_, 100. Shannon’s Entropy, 48. b_2_, 75. BMI/t_pp_	69. BMI/t_3_, 71. Weight/t_pi_, 74. Weight/t_pp_, 49. t_b2_, 59. Weight/∆T, 51. b_1_/a_1_, 46. b_1_, 47. t_b1_, 62. Weight/t_1_, 52. t_a1_/t_pp_, 66. BMI/t_2_, 67. Height/t_3_, 100. Shannon’s Entropy, 48. b_2_, 75. BMI/t_pp_

**Table 10:** Evaluation of the best performing algorithm for systolic blood pressure (SBP) and diastolic blood pressure (DBP).

Selection Criteria	Performance Criteria	Systolic Blood Pressure		Diastolic Blood Pressure	
		GPR	Ensemble Trees	GPR	Ensemble Trees
Features from the literature	MAE MSE RMSE R	12.27 240.25 15.50 0.71	12.68 246.74 15.70 0.71	8.31 96.90 9.84 0.62	8.82 109.92 10.45 0.54
All features (newly designed and from the literature)	MAE MSE RMSE R	12.06 272.32 16.50 0.70	12.95 316.71 17.80 0.59	7.70 97.31 9.86 0.63	8.31 110.87 10.53 0.57
ReliefF	MAE MSE RMSE R	**10.08** **219.08** **14.80** **0.74**	12.57 258.16 16.06 0.69	7.87 96.70 9.83 0.62	8.93 119.32 10.92 0.49
FSCMRMR	MAE MSE RMSE R	13.92 302.75 17.39 0.62	15.10 349.06 18.68 0.55	8.84 112.27 10.59 0.53	9.66 128.43 11.33 0.42
CFS	MAE MSE RMSE R	11.91 257.77 16.05 0.69	13.06 325.29 18.03 0.65	**7.64** **83.95** **9.16** **0.68**	8.27 103.70 10.18 0.58

**Table 11:** Evaluation of the outperforming algorithms for estimating SBP and DBP after optimization.

Selection Criteria	Performance Criteria	Systolic Blood Pressure		Diastolic Blood Pressure	
		Optimized GPR	Optimized Ensemble Trees	Optimized GPR	Optimized Ensemble Trees
Features from the literature	MAE MSE RMSE R	6.79 180.99 13.45 0.79	12.43 231.15 15.20 0.73	4.49 70.06 8.37 0.74	8.17 104.45 10.27 0.57
All features (newly designed and from the literature)	MAE MSE RMSE R	3.30 72.95 8.54 0.92	10.886 264.24 16.25 0.67	2.81 30.70 5.54 0.90	7.96 111.97 10.58 0.56
ReliefF	MAE MSE RMSE R	**3.02** **45.49** **6.74** **0.95**	11.32 284.69 16.84 0.65	**1.74** **12.89** **3.59** **0.96**	5.99 62.04 7.88 0.78
FSCMRMR	MAE MSE RMSE R	6.11 108.96 10.44 0.88	14.65 321.63 17.93 0.58	6.80 77.26 8.78 0.72	8.22 110.84 10.53 0.56
CFS	MAE MSE RMSE R	12.95 361.96 19.02 0.50	16.27 448.25 21.17 0.28	7.59 108.43 10.41 0.57	7.89 106.72 10.33 0.58

**Table 12:** Comparison with related works in relation to dataset, methodology, and estimation error.

Author	Method Used	Number of Subjects	Performance Criteria	Systolic Blood Pressure	Diastolic Blood Pressure
Kachuee et al. [24]	SVM	MIMIC II (1000 subjects)	MAE MSE RMSE R	12.38 - - -	6.34 - - -
Kim et al. [23]	Multiple nonlinear regression (MLP)	180 recordings, 45 subjects	MAE MSE RMSE R	5.67 - - -	- - - -
Kim et al. [23]	Artificial neural network (ANN)	180 recordings, 45 subjects	MAE MSE RMSE R	4.53 - - -	- - - -
Cattivelli et al. [25]	Proprietary algorithm	MIMIC database (34 recordings, 25 subjects)	MAE MSE RMSE R	- 70.05 - -	- 35.08 - -
Zhang et al. [27]	Support vector machine (SVM)	7000 samples from 32 patients	MAE MSE RMSE R	11.64 - - -	7.62 - - -
Zhang et al. [27]	Neural network (nine input neurons)	7000 samples from 32 patients	MAE MSE RMSE R	11.89 - - -	8.83 - - -
Zadi et al. [59]	Autoregressive moving average (ARMA) models	15 subjects	MAE MSE RMSE R	- - 6.49 -	- - 4.33 -
Slapničar et al. [30]	Deep learning (spectro-temporal ResNet)	MIMIC III database (510 subjects)	MAE MSE RMSE R	9.43 - - -	6.88 - - -
Su et al. [28] *	Deep learning (long short-term memory (LSTM))	84 subjects	MAE MSE RMSE R	- - 3.73 -	- - 2.43 -
This work	Gaussian process regression (GPR)	222 recordings, 126 subjects	MAE MSE RMSE R	**3.02** **45.49** **6.74** **0.95**	**1.74** **12.89** **3.59** **0.96**

* Deep learning algorithm on a small database.

**Table 13:** Comparison of this paper results with the Association for the Advancement of Medical Instrumentation (AAMI) standard.

		MEAN (mmHg)	SD (mmHg)	Subject
AAMI [62]	BP	≤5	≤8	≥85
This paper	SBP	3.02	9.29	126
	DBP	1.74	5.54	126

**Table 14:** Comparison of this paper results with the British Hypertension Society (BHS) standard.

		≤5 mmHg	≤10 mmHg	≤15 mmHg
BHS [63]	Grade A Grade B Grade C	60% 50% 40%	85% 75% 65%	95% 90% 85%
This paper	SBP DBP	69% 77%	76% 85%	92% 92%

## References

[1] Why is Blood Pressure Important. http://www.bloodpressureuk.org/microsites/u40/Home/facts/Whyitmatters/.

[2] 24-Hour Ambulatory Blood Pressure Monitoring (ABPM). http://www.bloodpressureuk.org/BloodPressureandyou/Medicaltests/24-hourtest/.

[3] Chowdhury M.E., Alzoubi K., Khandakar A., Khallifa R., Abouhasera R., Koubaa S., Ahmed R., Hasan A. (2019). Wearable real-time heart attack detection and warning system to reduce road accidents. Sensors.

[4] Chowdhury M.E., Khandakar A., Alzoubi K., Mansoor S., Tahir A.M., Reaz M.B.I., Al-Emadi N. (2019). Real-Time Smart-Digital Stethoscope System for Heart Diseases Monitoring. Sensors.

[5] Lee H., Kim E., Lee Y., Kim H., Lee J., Kim M., Yoo H.-J., Yoo S. (2018). Toward all-day wearable health monitoring: An ultralow-power, reflective organic pulse oximetry sensing patch. Sci. Adv..

[6] Chandrasekhar A., Kim C.-S., Naji M., Natarajan K., Hahn J.-O., Mukkamala R. (2018). Smartphone-based blood pressure monitoring via the oscillometric finger-pressing method. Sci. Transl. Med..

[7] Liang Y., Chen Z., Ward R., Elgendi M. (2018). Photoplethysmography and deep learning: Enhancing hypertension risk stratification. Biosensors.

[8] Elgendi M., Fletcher R., Liang Y., Howard N., Lovell N.H., Abbott D., Lim K., Ward R. (2019). The use of photoplethysmography for assessing hypertension. NPJ Digit. Med..

[9] Elgendi M. (2012). On the analysis of fingertip photoplethysmogram signals. Curr. Cardiol. Rev..

[10] Allen J. (2007). Photoplethysmography and its application in clinical physiological measurement. Physiol. Meas..

[11] Otsuka T., Kawada T., Katsumata M., Ibuki C. (2006). Utility of second derivative of the finger photoplethysmogram for the estimation of the risk of coronary heart disease in the general population. Circ. J..

[12] Millasseau S.C., Kelly R., Ritter J., Chowienczyk P. (2002). Determination of age-related increases in large artery stiffness by digital pulse contour analysis. Clin. Sci..

[13] Zheng Y., Poon C.C., Yan B.P., Lau J.Y. (2016). Pulse arrival time based cuff-less and 24-H wearable blood pressure monitoring and its diagnostic value in hypertension. J. Med. Syst..

[14] Lee C., Shin H.S., Lee M. (2011). Relations between ac-dc components and optical path length in photoplethysmography. J. Biomed. Opt..

[15] Utami N., Setiawan A.W., Zakaria H., Mengko T.R., Mengko R. Extracting blood flow parameters from Photoplethysmograph signals: A review. Proceedings of the 2013 3rd International Conference on Instrumentation, Communications, Information Technology and Biomedical Engineering (ICICI-BME).

[16] Bashkatov A., Genina E., Kochubey V., Tuchin V. (2005). Optical properties of human skin, subcutaneous and mucous tissues in the wavelength range from 400 to 2000 nm. J. Phys. D Appl. Phys..

[17] Van Gastel M., Stuijk S., de Haan G. (2016). New principle for measuring arterial blood oxygenation, enabling motion-robust remote monitoring. Sci. Rep..

[18] Liang Y., Elgendi M., Chen Z., Ward R. (2018). An optimal filter for short photoplethysmogram signals. Sci. Data.

[19] Waugh W., Allen J., Wightman J., Sims A.J., Beale T.A. (2018). Novel signal noise reduction method through cluster analysis, applied to photoplethysmography. Comput. Math. Methods Med..

[20] Lee H., Chung H., Ko H., Lee J. (2018). Wearable multichannel photoplethysmography framework for heart rate monitoring during intensive exercise. IEEE Sens. J..

[21] Xing X., Ma Z., Zhang M., Zhou Y., Dong W., Song M. (2019). An Unobtrusive and Calibration-free Blood pressure estimation Method using photoplethysmography and Biometrics. Sci. Rep..

[22] Rundo F., Ortis A., Battiato S., Conoci S. (2018). Advanced bio-inspired system for noninvasive cuff-less blood pressure estimation from physiological signal analysis. Computation.

[23] Kim J.Y., Cho B.H., Im S.M., Jeon M.J., Kim I.Y., Kim S.I. Comparative study on artificial neural network with multiple regressions for continuous estimation of blood pressure. Proceedings of the 2005 IEEE Engineering in Medicine and Biology 27th Annual Conference.

[24] Kachuee M., Kiani M.M., Mohammadzade H., Shabany M. Cuff-less high-accuracy calibration-free blood pressure estimation using pulse transit time. Proceedings of the 2015 IEEE International Symposium on Circuits and Systems (ISCAS).

[25] Cattivelli F.S., Garudadri H. Noninvasive cuffless estimation of blood pressure from pulse arrival time and heart rate with adaptive calibration. Proceedings of the 2009 Sixth International Workshop on Wearable and Implantable Body Sensor Networks.

[26] Xing X., Sun M. (2016). Optical blood pressure estimation with photoplethysmography and FFT-based neural networks. Biomed. Opt. Express.

[27] Zhang Y., Feng Z. A SVM method for continuous blood pressure estimation from a PPG signal. Proceedings of the 9th International Conference on Machine Learning and Computing.

[28] Su P., Ding X.-R., Zhang Y.-T., Liu J., Miao F., Zhao N. Long-term blood pressure prediction with deep recurrent neural networks. Proceedings of the 2018 IEEE EMBS International Conference on Biomedical & Health Informatics (BHI).

[29] Gotlibovych I., Crawford S., Goyal D., Liu J., Kerem Y., Benaron D., Yilmaz D., Marcus G., Li Y. (2018). End-to-end deep learning from raw sensor data: Atrial fibrillation detection using wearables. arXiv.

[30] Slapničar G., Mlakar N., Luštrek M. (2019). Blood Pressure Estimation from Photoplethysmogram Using a Spectro-Temporal Deep Neural Network. Sensors.

[31] Liang G.L.Y., Chen Z., Elgendi M. (2018). PPG-BP Database. https://figshare.com/articles/PPG-BP_Database_zip/5459299/.

[32] Liang Y., Chen Z., Liu G., Elgendi M. (2018). A new, short-recorded photoplethysmogram dataset for blood pressure monitoring in China. Sci. Data.

[33] Liang Y., Chen Z., Ward R., Elgendi M. (2018). Hypertension assessment via ECG and PPG signals: An evaluation using MIMIC database. Diagnostics.

[34] Liang Y., Chen Z., Ward R., Elgendi M. (2019). Hypertension assessment using photoplethysmography: A risk stratification approach. J. Clin. Med..

[35] Ferdinando H., Huotari M., Myllylä T. Photoplethysmography signal analysis to assess obesity, age group and hypertension. Proceedings of the 2019 41st Annual International Conference of the IEEE Engineering in Medicine and Biology Society (EMBC).

[36] Kavsaoğlu A.R., Polat K., Hariharan M. (2015). Non-invasive prediction of hemoglobin level using machine learning techniques with the PPG signal’s characteristics features. Appl. Soft Comput..

[37] Elgendi M., Norton I., Brearley M., Abbott D., Schuurmans D. (2014). Detection of a and b waves in the acceleration photoplethysmogram. Biomed. Eng. Online.

[38] Kavsaoğlu A.R., Polat K., Bozkurt M.R. (2014). A novel feature ranking algorithm for biometric recognition with PPG signals. Comput. Biol. Med..

[39] Mahbub Z.B., Rabbani K. (2007). Frequency domain analysis to identify neurological disorders from evoked EMG responses. J. Biol. Phys..

[40] Yang S., Zhang Y., Cho S.-Y., Morgan S.P., Correia R., Wen L. Cuff-less blood pressure measurement using fingertip photoplethysmogram signals and physiological characteristics. Proceedings of the Optics in Health Care and Biomedical Optics VIII.

[41] Chatterjee A., Roy U.K. PPG Based Heart Rate Algorithm Improvement with Butterworth IIR Filter and Savitzky-Golay FIR Filter. Proceedings of the 2018 2nd International Conference on Electronics, Materials Engineering & Nano-Technology (IEMENTech).

[42] Sun S., Peeters W.H., Bezemer R., Long X., Paulussen I., Aarts R.M., Noordergraaf G.J. (2019). Finger and forehead photoplethysmography-derived pulse-pressure variation and the benefits of baseline correction. J. Clin. Monit. Comput..

[43] Maxwell J.C. (1881). A Treatise on Electricity and Magnetism.

[44] McDuff D., Gontarek S., Picard R.W. (2014). Remote detection of photoplethysmographic systolic and diastolic peaks using a digital camera. IEEE Trans. Biomed. Eng..

[45] Laurin A. BP_Annotate. https://www.mathworks.com/matlabcentral/fileexchange/60172-bp_annotate/.

[46] Pan J., Tompkins W.J. (1985). A real-time QRS detection algorithm. IEEE Trans. Biomed. Eng..

[47] Sun J., Reisner A., Mark R. A signal abnormality index for arterial blood pressure waveforms. Proceedings of the 2006 Computers in Cardiology.

[48] Monte-Moreno E. (2011). Non-invasive estimate of blood glucose and blood pressure from a photoplethysmograph by means of machine learning techniques. Artif. Intell. Med..

[49] Kurylyak Y., Lamonaca F., Grimaldi D. A Neural Network-based method for continuous blood pressure estimation from a PPG signal. Proceedings of the 2013 IEEE International Instrumentation and Measurement Technology Conference (I2MTC).

[50] Kira K., Rendell L.A. (1992). The feature selection problem: Traditional methods and a new algorithm. Aaai.

[51] Kononenko I., Šimec E., Robnik-Šikonja M. (1997). Overcoming the myopia of inductive learning algorithms with RELIEFF. Appl. Intell..

[52] Roffo G. (2017). Ranking to learn and learning to rank: On the role of ranking in pattern recognition applications. arXiv.

[53] Ding C., Peng H. (2005). Minimum redundancy feature selection from microarray gene expression data. J. Bioinform. Comput. Biol..

[54] Darbellay G.A., Vajda I. (1999). Estimation of the information by an adaptive partitioning of the observation space. IEEE Trans. Inf. Theory.

[55] Khandakar A., Chowdhury M.E.H., Kazi M.K., Benhmed K., Touati F., Al-Hitmi M., Antonio S.P. (2019). Gonzales. Machine learning based photovoltaics (PV) power prediction using different environmental parameters of Qatar. Energies.

[56] Sit H. Quick Start to Gaussian Process Regression. https://towardsdatascience.com/quick-start-to-gaussian-process-regression-36d838810319/.

[57] Ensemble Algorithms. https://www.mathworks.com/help/stats/ensemble-algorithms.html/.

[58] Filion A. (1994–2020). Applied Machine Learning, Part 3: Hyperparameter Optimization. https://www.mathworks.com/videos/applied-machine-learning-part-3-hyperparameter-optimization-1547849445386.html/.

[59] Zadi A.S., Alex R., Zhang R., Watenpaugh D.E., Behbehani K. (2018). Arterial blood pressure feature estimation using photoplethysmography. Comput. Biol. Med..

[60] Stergiou G.S., Alpert B., Mieke S., Asmar R., Atkins N., Eckert S., Frick G., Friedman B. (2018). A universal standard for the validation of blood pressure measuring devices: Association for the Advancement of Medical Instrumentation/European Society of Hypertension/International Organization for Standardization (AAMI/ESH/ISO) Collaboration Statement. Hypertension.

[61] Mousavi S.S., Firouzmand M., Charmi M., Hemmati M., Moghadam M., Ghorbani Y. (2019). Blood pressure estimation from appropriate and inappropriate PPG signals using A whole-based method. Biomed. Signal Process. Control.

[62] Association for the Advancement of Medical Instrumentation (2003). American National Standard. Manual, Electronic or Automated Sphygmomanometers.

[63] O’brien E., Waeber B., Parati G., Staessen J., Myers M.G. (2001). Blood pressure measuring devices: Recommendations of the European Society of Hypertension. BMJ.

